# Effects of early feeding technologies providing methionine supplementation on performance, lipid oxidation, and some immune-related gene expression in broiler chicken

**DOI:** 10.1016/j.psj.2025.105335

**Published:** 2025-05-26

**Authors:** Virág Ács, Nóra Szeli, József Nagy, Szilvia Áprily, Annamária Tischler, Orsolya Csötönyi, Ildikó Jócsák, Ildikó Benedek, Örs Petneházy, Janka Turbók, Judit Enyezdi, Veronika Halas

**Affiliations:** a HUN-REN-MATE Mycotoxins in the Food Chain Research Group, Guba Sándor Street, Kaposvár H-7400, Hungary; b Hungarian University of Agriculture and Life Sciences, Kaposvár Campus, Kaposvár H-7400, Hungary; c AVI-VET Ltd., Kaposvár H-7400, Hungary; d Medicopus Nonprofit Ltd., Kaposvár H-7400, Hungary; e Animal Health Diagnostic Department, National Food Chain Safety Office, Animal Health Diagnostic Directorate, Kaposvár H-7400, Hungary; f Pathodiagnostica Ltd., Pécs H-7623, Hungary

**Keywords:** *In-ovo* nutrition, Hydrogel, Lipid oxidation, Gene expression, Broiler

## Abstract

*In ovo* administration of DL-methionine and post-hatch Hydrogel® supplements were tested to examine the impact of early feeding on performance and immune-related traits in a commercial broiler stock. One thousand one hundred and twenty Ross 308 eggs were incubated and assigned to seven treatment groups: intact (no *in ovo* administration) and immediate feed access (**C1**), *in ovo* saline treatment and immediate feed access (**C2)**, intact and delayed feeding (**ID**), *in ovo* saline treatment and delayed feeding (**IoS**), *in ovo* DL-Methionine treatment and delayed feeding (**IoM**), intact and delayed access to feed, but immediate access to commercial Hydrogel® without (**Hyd**) or with 5mg/kg (**HydM**) DL-methionine post-hatch. The results showed, that the *in-ovo* methionine may have positive effects on the weight gain of the birds (*p* < 0.001) compared to the commercial Hydrogel® however, it cannot compete with the immediate feeding. The number of heterophils decreased significantly (*p* < 0.001) by day 21 in **ID** and **IoS** compared to the immediately fed control (**C1**). The number of lymphocytes, monocytes, and eosinophils, increased in treatments supplemented with methionine (*p* < 0.05) *(****IoM****,****HydM)*** indicating enhanced immune protection.

There were no differences in the total antioxidant capacity (**FRAP**) and malonaldehyde concentration (**MDA**) (*p* = 0.07) in the examined groups. The Cytochrome P450 H1 (**CYP2H1**) gene was downregulated in all treatment groups (on days 21 and 35) indicating a slower metabolism, particularly in the ID group compared to C1 and C2 (*p* < 0.001). The **HydM** treatment could upregulate the **IL2** expression as the immediate feeding, while only **IoM** treatment resulted in significant downregulation by day 35 (*p* < 0.001). **IL6** was upregulated in all treatment groups (*p* < 0.001) except for **HydM,** where the gene expression did not differ from the housekeeping gene. Early administration of dietary methionine has a positive effect on performance and the immune system, however, none of the early feeding methods can compete with immediate feed access. The possible positive effects of early nutrition and its epigenetic impact should be examined in further studies.

## Introduction

With the growth of the human population, a huge demand for meat production makes broiler production more intensive than other livestock species (Bessei, 2018). This phenomenon has been particularly true for the past few decades, which projects further genetic selection for growth rate, and meat quality traits (Korver, 2023). On the other hand, this rapid development cannot be continued without considering the animals' general health (Bradshaw et al., 2002). Furthermore, the call for eliminating growth-promoting antibiotics from poultry feed may inspire researchers to improve health and boost immune defense through dietary supplements (Yaquoob et al., 2022) and particularly early feeding.

Incubation conditions and the neonatal phase are critical as the chick shifts from endogenous nutrient utilization to exogenous (Ferket, 2012; Patricia et al., 2020). The nutrient reserves of the egg are limited; thus, the embryos – particularly those of intensive genotype – may need supplements during embryogenesis (Uni et al., 2012) or immediate feeding post-hatch. The performance along with the development of the gastrointestinal tract (**GIT**), and the microbiome are also affected by the 36-72 h lag-time after pipping (Das et al., 2021; Kpodo, 2023).

Several studies reported, that short-time feed deprivation (lasting no longer than 24 h) does not significantly affect the long-term immune competence or the performance of broilers (Bhanja et al., 2009; Lamot et al., 2014). However, in commercial practice, birds often experience prolonged feed deprivation, sometimes extending to 48 h or more, which relies on yolk sac reserves. This extended fasting period can adversely impact the birds' immune systems over time and may alter cell signaling, including the functions of protein mediators such as cytokines. According to a comprehensive meta-analysis by de Jong (2017), longer periods of post-hatch feed deprivation are associated with significantly lower body weight and a higher total mortality rate. Additionally, delayed nutrient uptake affects the utilization of the yolk sac, leading to impaired gut development and compromised immune functions (Dibner et al., 1998; Bhanja, 2008).

When young birds are exposed to stress (e.g., starvation during transport), it may cause behavioral or physiological changes (Etches et al., 1995). These cellular stress reactions can be measured by the gene expression of some xenobiotic enzymes (CYP 1-3 superfamily) or via the ferric-reducing ability of plasma (**FRAP**) and the malonaldehyde (**MDA**) concentration of the blood, which is overproduced during oxidative stress (Gawel et al., 2004). Oxidative stress interferes with the immune competence of the birds, and feed deprivation may have further epigenetic impact. Despite the view of a few years ago that the avian immune system is fully developed by the late embryonic phase (Bar-Shira and Friedman, 2006; Reemers et al., 2010), recently it has been confirmed that its maturation takes up to 30-34 days post-hatch (Fellah et al., 2014). For that reason, dietary restrictions can lead to impaired systemic immune competence as the thymus and the gut-associated lymphoid tissue (GALT) are very sensitive to the duration of feed deprivation (Klasing, 2007). Dietary amino acids can assist in the repair of intestinal mucosa as well as altering gene expression in autoinflammatory processes (Wu et al., 2009). Methionine (Met), as often the first limiting amino acid in broiler nutrition, plays a pivotal role in the humoral and cellular immune responses in poultry (Swain and Jhori, 2000; Attia et al., 2005), and participates in immune cell synthesis thus increases the antibody content of the blood (Tsiagbe, 1987). Sufficient dietary Met is indispensable for cytokine production like the serum IL-2 and T-cell proliferation (Wu et al., 2018). It has been confirmed that methionine-deficient feed leads to lower egg weight and hatchability (Liu et al., 2022). However, it is unclear whether the low hatchability is due to the smaller egg weight or if it results from some specific issues related to the lack of sulphur containing amino acids.

Recently it was confirmed that perinatal, *in ovo* methionine supplements may enhance the birds' general health and antioxidant status (Lugata et al., 2024; Tombarkiewitz et al., 2024) by reducing oxidative stress (Ruth and Field, 2013). For nearly two decades, the poultry sector has implemented early nutrition methods such as gel supplements (provided post-hatch) or injecting substances into the egg for the embryo (pre-hatch) to enhance the performance and boost the immunity of the birds. The relevant studies report on the detrimental effect of delayed access to feed or evaluate the impact of early feeding. However, only a few research compares early feeding methods to immediate feeding (Arevan et al., 2023). These findings are still preliminary, and require further investigation. To fully understand the benefits of early feeding, more comparative studies are needed. Furthermore, the mechanisms of how early nutrition affects the immune system at the molecular genetic level remain incompletely understood.

For these reasons, growth performance as well as expression of interleukin 2- and 6 **(IL-2 and IL6**) and Cytochrome P450 H1 (**CYP2H1**) genes were examined along with serum antioxidant capacity to investigate the physiological, genetic, and metabolic changes of a commercial broiler stock to early nutrition technologies providing methionine supplementation.

## Materials and methods

The experiment was conducted at the Hungarian University of Agricultural and Life Sciences (**MATE**) Kaposvár Campus, Department of Farm Animal Nutrition by the Declaration of the Hungarian National Scientific Ethical Committee of Animal Experimentation for studies involving animals: protocol license number: SO/31/00956/2020.

### Egg incubation and experimental design

The study included 1120 Ross 308 broiler eggs from Aviagen Ltd. (Hungary) without disinfection treatments and held in transport boxes under 20°C for 6 days without rotation or extra humidification due to the short storage time. A PLM B1350 two-staged incubator (Tárnok, Hungary) was used with 9 tray levels for the incubation period. Each level contained an among-level mobile ventilation, humidity, and temperature measurement system. The hatching protocol was carried out according to the Aviagen Ross 308 Broiler Management Handbook (Aviagen, 2019) recommendations: the dry bulb temperature and humidity were set at 37.9 ± 0.1°C and 65 ± 3 %, respectively all the way through incubation. The eggs were candled on day 10 and 17 to exclude infertile eggs or dead embryos. The fertility of eggs was calculated after candling on day 17 according to Alasahan et al. (2016) by the following formula:

Fertility rate % = number of fertile eggs/total number of eggs set * 100

All eggs were weighted and randomly assigned into 7 treatment groups (Table 1). A balanced incomplete block design was implemented (3 treatments, 2 blocks), as in ovo methionine treatment is not commonly applied along with immediate feeding in practice. The treatment groups were formed as follows: **C1**: intact eggs (no *in ovo* treatment) and as hatched chicks with immediate feed access, used as control group; **C2**: incubated eggs injected with saline (0.9 g/mL concentration of NaCl) and as hatched chicks with immediate feed access - to investigate the effect of *in ovo* intervention per se, **ID**: intact eggs and as hatches chicks with 48 h delayed access to feed – used as negative control. **IoS**: *in-ovo* saline treatment and delayed feed access. **IoM**: *in ovo* methionine (0.5 g/mL) supplementation (VWR International Hungary Ltd, Debrecen) - dissolved in physiological saline and delayed feed access post-hatch. **Hyd**: no *in ovo* intervention (control) and chicks had delayed feed access, but access to Hydrogel® (Bábolna Feed Ltd., Nagyigmánd, Hungary) supplement in the transport boxes, **HydM**: no *in ovo* intervention (control), delayed feed access, but access to Hydrogel® with 0.5 % methionine enrichment respectively. The composition of the commercial Hydrogel® can be found in Table 2.

**Table 1:** Definitions of treatment groups in the study.

Treatment code	Feed access	Early nutrition method	Methionine supplement
C1	Immediate	-	-
C2		*in-ovo*, saline	-
ID	Delayed (48 h)	-	-
IoS		*in-ovo*, saline	-
IoM		*in-ovo*, DL-Met	+
Hyd		post-hatch gel*	-
HydM		post-hatch gel*	+

C1: intact control group, with immediate feed access after hatching; C2: control group injected with *in-ovo* saline (0.9 g/mL concentration of NaCl) and immediate feed access after hatch; ID: intact with delayed feed access by 48 hrs; IoS: *in-ovo* saline and delayed feed access; IoM *in-ovo* methionine (0.5 g/mL)- dissolved in physiological saline- and delayed feed access. Hyd: delayed feed access with Hydrogel®, HydM: delayed feed access with Hydrogel® and methionine.

⁎ gel: Hydrogel with (+) or without (-)DL- methionine.

**Table 2:** Composition of Hydrogel® as given by the producer.

DM	%	5
ME	MJ/kg dry matter	5.4
Corn starch	%	30
Vitamin C	mg/kg	5000
Pediococcus acidilactici (E1712)	CFU/g	1 × 10^10^

DM: dry matter; ME: metabolizable energy.

All eggs were placed on 9 tray levels, where 320 eggs were planned to be injected with *in ovo* saline and 160 eggs were planned to be injected with *in ovo* DL-methionine while the rest of the eggs remained intact.

Right before the *in-ovo* intervention, after the second candling on day 17, the treatment groups were equalized, (to 143 eggs in all treatment groups) as 116 eggs were bloody or infertile and excluded from the experiment.

Then, injected and non-injected eggs were separately and randomly placed back to the hatchery.

After the chicks were removed from the hatchery incubator, their sex was identified based on the growth length of the wing feathers. To ensure accurate tracking of individual body weight **(BW)** and average daily gain **(ADG**), each chick was assigned a permanent wing tag with a unique five-digit number, and then their body weight was recorded. The birds were placed in paper boxes in groups of 25, organized according to their treatment groups. Birds in groups 1 and 2 (C1 and C2) were immediately settled in the barn and provided with feed and water. In contrast, the remaining groups were kept in a room with a controlled temperature for 48 h before being moved to their respective environments. During this 48-hour delay, birds in groups ID, IoS***,*** and IoM had no access to feed or water, while Hyd and HydM were provided with Hydrogel® (Table 2), either without or with methionine enrichment. Hydrogel® consisted of corn starch, dextrose, and probiotic lactic acid bacteria with or without a 5 g/kg DL-*Met* supplement for HydM group.

### *In ovo* intervention

The *in-ovo* injection was carried out with a 2 mL syringe with a 21-gauge needle following the method described by Uni and Ferket (2004) on day 17 of incubation. To prevent microbiological contamination the injection procedure took place in a sterile cabinet (ScanLaf, LaboGen Inc., Lillerød Denmark). All eggs were cleaned with cotton wool soaked in an iodine solution. The eggs were carefully drilled on the blunt end through the air chamber without reaching the shell membrane. Prior to the injection, the embryo position was checked. After this, 0.5 mL of the solution was transferred with amniotic inoculation. To eliminate the risks of pathogen entry, a sterile, plastic tape was applied, and the eggs were placed back into the incubator until day 21, when hatching occurred. Due to the *in ovo* intervention the eggs were out of the hatchery for max. of 30 min.

### Feeding management and housing

The birds were weighed with 0.1 g precision on hatching day, and on day 3 (end of feed deprivation) to measure hatching weight and weight loss, and randomly placed into floor pens (16 birds/pen, 7 pens/treatment). To ensure uniform stocking density, the remaining birds were not used in the trial, unless dead birds in the first week had to be replaced. Each pen represented an individual treatment group. The installation was set up in compliance with EU regulations for temperature, humidity, air movement, harmful gas, dust concentration, hours of light, and intensity requirements of livestock, according to the recommendations of Aviagen (2019).

The live weight of the birds was also measured with 0.1 g precision on days 10, 21, and 35. The birds were fed *ad libitum* from self-feeders during the trial. One feeder was presented per pen providing enough space for all birds in the pen. Drinking water was also available *ad libitum*. A three-phase feeding program was applied by the following method: day 1-10 starter ration (crumbled feed); day 11-21, grower feed; and day 22–35 finisher feed. In the first week a commercial crumble, and later a pelleted feed was offered which was produced by Agroszász Ltd. (Szászvár, Hungary). The composition of the offered feed is demonstrated in Table 3.

**Table 3:** The composition of the feed.

Ingredients	Starter (1–10)		Grower (11–21)		Finisher (22–35)
Corn (grain)	551		577		601
Corn gluten (60 %)	32		32		32
Sunflower meal	53.5		53.5		75
Soybean meal (CP 44.2 %)	262		230		175
Fat, vegetable	44.7		55		67.00
MCP	18.7		17.5		15
Limestone	15		13.5		12.2
NaCl	2.7		2.7		2.7
L-Lysin HCl	5.2		4.6		4.3
DL-Methionine	4.5		3.9		3.2
L-Threonine	2.6		2.3		1.8
Premix^1^	5.00		5.00		5.00
**Total**	**1000.00**		**1000.00**		**1000.00**
**Nutrient content g/kg)**					
AMEn (MJ/kg)	12.5		12.9		13.4
DM %	90		91.3		91.1
Crude protein	204.2		190.7		174.9
Crude fat	71.87		82.3		94.4
Crude fiber	41.5		41.1		44.8
Lysine*	13.5		12,1		10,8
*M* + C*	10.8		9.9		9.0
Threonine*	9.7		8,8		7,8
Tryptophan*	2.4		2.3		1.7
Ca	9.6		8.7		7.8
P _available*_	4.7		4.5		3.9
Na*	1.7		1.7		1.7

1 Premix feed contents per kilogram: Zn: 22,032 mg, Cu: 3200 mg, Fe: 16,020 mg, Mn: 21,948 mg, I: 300 mg, Se: 70 mg, Co: 20 mg, Vit. A: 324,0000 IU, Vit. D3: 810,000 IU, Vit. E: 25,800 IU, Vit K3: 810 mg, Vit. B1: 810 mg, Vit. B2: 1890 mg, Vit. B3: 10,800 mg, Vit. B5: 3240 mg, Vit. B6: 1350 mg, Vit B12: 6.8 mg, Folic acid: 270 mg, Biotin: 32 mg.

⁎ Represent calculated values.

Each feed was formulated on a corn-soybean meal basis. Nutritional content - dry matter, crude protein, fat, ash, calcium, and phosphorus - was determined by the University Lab Center of MATE according to the recommendations of the Association of Official Analytical Chemists (Official Method of Analysis, 2012).

Feed intake (FI) and feed conversion ratio (FCR) were recorded by measuring the offered, and remaining feed for each phase per pen.

### Blood samples and blood smears

Blood samples were collected from 10 birds/treatment from the *vena jugularis*, and collected into 5 ml blood collection tubes with anticoagulants (**EDTA**) for further evaluation. Blood smears were prepared immediately after the collection process, and a Natt-Herrick’s solution (Schalm et al., 1975) was used to determine the relative number of different leukocytes. Three images were captured per slide with a BX43 Olympus light microscope and an Olympus DP23 digital camera (Olympus, Japan). The number of eosinophils and heterophils, lymphocytes, basophils, and monocytes were counted per 100 white blood cells per image and the average percentages were calculated.

After that, the absolute number of blood cells was calculated by Douglas et al. (2010) by the following formula:

Leukocyte number (Douglas et al., 2010) * Relative number of the white blood cells/100

Plasma was obtained at 4°C for 12 min in a Hettich 220R centrifuge (1500 g = 3760 RPM). After that, 0.5 ml blood was pipetted to another tube for alikvotation and stored at −80°C to determine oxidative capacity and lipid oxidation.

### Antioxidative capacity and lipid oxidation

The ferric-reducing ability of plasma (**FRAP**) was carried out by the recommendations of Benzie and Strain (1996). The method is based on the oxidized form of reducing Fe^3+^ to Fe^2−^ under acidic conditions, by a blue-colored ferrous–tripyridyltriazine complex. The intensity of the color change determines the antioxidant capacity. The assay consists of solutions containing the following ingredients of stock solutions:

• Solution A: 300 mM Na-acetate buffer (500 mL solution: 1,55 g Na–acetate 3 H_2_O + 8 mL acetic acid, distilled water),
• Solution B: 10 mM TPTZ (2,4,6-tripiridyle-s-triazine) 40 mM/L dissolved in HCl
• Solution C: 20 mM ferric-chloride (FeCl_3×_6H_2_O) 54 mg, 10 mL dissolved in distilled water
• The FRAP working reagent consists of the solutions mentioned above, in the following way: A: B: *C* = 10:1:1.

Blood samples were slowly thawed on ice blocks from −80°C to room temperature, and 100 µL of blood plasma + 2.9 mL of FRAP reagent were mixed. Then the samples have been incubated at 37 °C for 4 min, after, the absorbance was measured at 593 nm (SmartSpec™ Plus spectrophotometer 1000 Hercules, CA). The results were given in ascorbic acid equivalent values. Absorbance values were represented as concentration values, and a calibration line was created for the parameters with unknown antioxidant capacity.

MDA content was determined by the thiobarbituric acid (TBA) reaction with modifications of the original method of Heath and Packer (1968). For each sample, 0.25 mL of blood plasma was pipetted into a reaction tube containing 1.00 mL of working solution (20 % trichloroacetic acid (TCA) solution containing 0.5 % TBA (w/v) as described by Oszlányi et al. (2020). The total reaction volume was therefore 1.25 mL per sample. The mixture was thoroughly vortexed to ensure homogeneity. Reaction solutions were incubated in a water bath (Julabo ED-5 M, JULABO GmbH Gerd-Juchheim-Strasse 1 77960 Seelbach / Germany) for 30 min at 96 °C, then were stopped by immediate cooling on ice and were subsequently centrifuged at 10,000 rpm for 5 min.

Absorbance (532 and 600 nm) at was recorded using a SmartSpec™ Plus spectrophotometer (1000 Alfred Nobel Drive Hercules, CA), and MDA concentration was calculated by subtracting the non-specific absorption at 600 nm from the absorption at 532 nm using an absorbance coefficient of extinction, 156 mM^−1^ cm^−1^. The results were expressed as nmol mL^−1^.

### cDNA isolation and quantitative reverse transcription PCR (RT-qPCR)

Four chickens from each treatment group were euthanized with 70 % CO_2_ for sample collection on days 21 and 35 of the study for molecular genetic measurements. Expression of IL-2, IL-6, and CYP450 2H1 genes were examined on days 21 and 35 from the liver (tissue sample size was 3 mm x 3 mm). For the immediate preservation of the gene expression pattern for reliable gene expression analysis, samples were placed into 15 mg RNAlater® (Thermofisher Scientific, Waltham, MA). Total RNA was extracted from each sample using RNeasy® Mini Kit (250) (QIAGEN Sciences, Hilden, Germany) according to the manufacturer's recommendations. For stabilized samples, tissues were carefully removed from RNAlater® using heat sterilized (220 °C) forceps and immediately transferred into appropriately sized lysis vessels at room temperature (approximately 20–25 °C). Disruption and homogenization were performed in Buffer RLT supplemented with 1 % β-mercaptoethanol to ensure efficient cell lysis and RNase inactivation. During tissue processing, the samples were homogenized by TissueLyser II (QIAGEN Sciences, Hilden, Germany) at 5 s with 23.5 1/s frequency, in 600 μL lysis buffer (RLT) in order to create a homogenous tissue lysate.

Following complete homogenization, lysates were centrifuged in a Hettich Micro 220 / 220 R microcentrifuge (Andreas Hettich Ltd., Tuttlingen**,** Germany) at maximum speed (≥12,000 × g) for 3 min to remove residual tissue fragments. The cleared supernatant was then mixed in a 1:1 ratio with 70 % ethanol, creating binding conditions that facilitate selective adsorption of RNA molecules ≥200 nucleotides to the silica-based membrane within the RNeasy® spin columns. The lysate-ethanol mixture was applied to the columns in aliquots up to 700 µL, followed by centrifugation to allow RNA binding.

Subsequently, membrane-bound RNA was subjected to a two-step washing procedure using Buffer RW1 (which removes proteins and carbohydrates) and Buffer RPE (which eliminates salts and other residual contaminants). To ensure the complete removal of ethanol from the column membrane an extended drying spin (2 min) was included after the final RPE wash. RNA was eluted in 30 µL of RNase-free water, directly applied to the membrane, and incubated for 1 min before centrifugation.

The concentration and purity of the recovered RNA were assessed spectrophotometrically using a NanoDrop™ One instrument (Thermo Fisher Scientific, Waltham, MA). RNA samples with A260/A280 ratios between 1.8 and 2.1 and A260/A230 values above 1.8 were considered of sufficient purity and used for downstream applications. For each sample, equal amounts of RNA (100 ng) were used to synthesize cDNA using the QuantiTect® Reverse Transcription Kit (QIAGEN Sciences, Hilden, Germany), the synthesis reaction was allowed to run for 15 min at 42°C, after that 3 min at 95°C and the cDNA was stored at −80°C or used immediately. and stored at −80°C for further processing. After collection, the tissues were homogenized by TissueLyser II (QIAGEN Sciences, Hilden, Germany) at 5 s with 23.5 1/s frequency, in 600 μl lysis buffer (**RLT**). Total RNA was extracted from each sample using RNeasy® Mini Kit (250) (QIAGEN Sciences, Hilden, Germany) according to the manufacturer's recommendations. RNA quantity and quality were determined using a NanoDrop™ One spectrophotometer (Thermo Fisher Scientific, Waltham, MA). For each sample, equal amounts of RNA (100 ng) were used to synthesize cDNA using the QuantiTect® Reverse Transcription Kit (QIAGEN Sciences, Hilden, Germany), the synthesis reaction was allowed to run for 15 min at 42°C, after that 3 min at 95°C and the cDNA was stored at −80°C or used immediately.

The primer design was set according to Yarru et al. (2009) as follows (Table 4).

**Table 4:** Primer design according to Yarru et al. (2009).

Primer	Sequence
**Cytochrome P450 (CYP2H1)**	For- 5′ATCCCCATCATTGGAAATGT3′ Rev-5′TCGTAGCCATACAGCACCAC3′
**Interleukin 2 (IL2)**	Rev- 5′TGCAGTGTTACCTGGGAGAA For- 5′CTTGCATTCACTTCCGGTGT
**Interleukin 6 (IL6)**	Rev- 5′GACTCGTCCGGAGAGGTTG For- 5′CGCACACGGTGAACTTCTT

The qPCR reaction mix consisted of 5 μl of 2 × qPCRBIO SyGreen Mastermix (PCR Biosystems, London, UK), 0.3 μl of each primer pair (10 μM) and 1 μl cDNA template and 3.4 μl distilled water in the 10 μl reaction volume. The PCR was performed on a Stratagene Mx3000P thermocycler (Agilent Technologies, Santa Clara, CA), the amplification procedure consisted of the following steps: 10 min at 95°C followed by 60 cycles of 30 s at 95°C and 1 min at 60°C and 50 sec 72°C. Amplification specificity was performed by melting curve analysis.

### Statistical analysis

The data were analyzed as an incomplete randomized block design with 7 treatments. A Shapiro–Wilk normality test was conducted to assess the normality of the data. Since the hatching data exhibited a skewed distribution, a Kruskal–Wallis test was performed to evaluate the effects of in-ovo treatments. The statistical grouping was based on whether the eggs received an in-ovo treatment. The groups were classified as follows: C1, ID, Hyd, and HydM were “intact” groups, while C2 and IoS were “treated in-ovo with saline,” and IoM was “treated with in-ovo methionine.” Total hatchability was calculated using the following formula:

Hatchability (%) = Hatched eggs / Total number of eggs put in the incubator *100

Where the weak and suffocated chicks did not count as “hatched eggs”. For performance, antioxidant capacity, and leukocyte data, Levene’s test was used to examine group homogeneity among treatment groups. After that, a multi-way ANOVA was applied with the effect of treatment and sex on live weights and ADG at different ages as these traits were measured individually. Thereafter, a one-way ANOVA was applied for FI, FCR, leukocyte cell number, FRAP, and MDA levels.

The results of the qPCR data were firstly analyzed by the method of Livak and Schmittgen (2001), where relative gene expression of the examined genes (CYP2H1, IL2, and IL6) was calculated compared to a housekeeping gene (**GADPH**) as the level of gene expression of this reference gene remains very stable to any treatments (Dheda et al., 2004). Then, the 2ˆ-ΔΔCt method (Mutch et al., 2002) was used to calculate the Fold change values (FC) of the examined genes with the correction of Rao et al. (2013) called the individual efficiency-corrected method by the following formula:

$$FC=\frac{\frac{X_{0,D}}{X_{0,B}}}{\frac{X_{0,C}}{X_{0,A}}}=\frac{\frac{{\left(E_{B}\right)}^{CT\prime B}\left(EB-1\right)}{{\left(E_{D}\right)}^{CT\prime D}\left(ED-1\right)}}{\frac{{\left(E_{A}\right)}^{CT\prime A}\left(EA-1\right)}{{\left(E_{C}\right)}^{CT\prime C}\left(EC-1\right)}}$$

Where X_0_ is the initial DNA amount, E is the amplification efficiency, A, B, C, and D refer to the combinations of samples in the experimental design, and CT’ is the threshold cycle.

Then, we applied a robust between-sample normalization on the FC data based on (Love et al., 2014), by log^2^ transformation for data symmetry and to get the relative log-fold-change values compared to the housekeeping gene to see which genes are the up-and down-regulated depending on the treatment.

Moreover, to mitigate the false discovery rate (**FDR**) associated with differentially expressed genes, we utilized bootstrapping on the base data, an essential step given the constraints of our small sample size (Meuwissen and Goddard, 2004). To further refine our results, we employed the bias-corrected accelerated (BCa) bootstrap interval, addressing any bias and skewness in the distribution of our bootstrap estimates (Chen and Fritz, 2021). Ultimately, we fitted a generalized linear model (GLM) to our data as described by Kerr and Churchill (2001), and the model formula is presented as follows:

$$y_{\text{ijk}}=\mu +m_{i}+g_{j}+t_{k}+{\left(g*t\right)}_{\text{jk}}+A$$

Where Y was the relative expression of the gene in question (2^–ΔΔCt^) as ΔΔC_t_ =ΔC_t treated_-ΔC_t intact_ and ΔC_t_ = (C_t target gene_-C_t reference gene)_, μ is the overall mean, m_i_ is the effect of the i^th^ sample, g_j_ is fixed effect of the treatment, t_k_ is fixed effect of the sex and A is the additive genetic effect according to the great individual variance. The statistical analysis was carried out with SPSS 29.0.2.0 (IBM Corp., 2023).

## Results

### Hatchability results

**Fig. 1:**
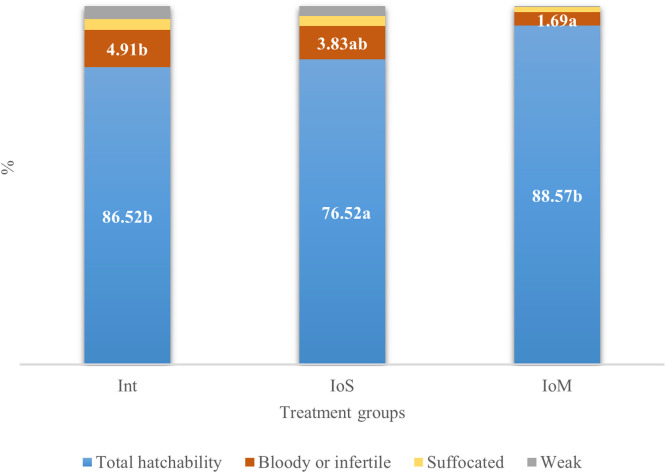
The hatching rate and proportions of dead chicks at hatch. The intact group represents all the treatments where no *in-ovo* intervention occurred (**C1, Int, Hyd, HydM**).

In our study, the hatching rate was satisfactory. Overall, 6.1 % of the hatchlings were either weak or suffocated at hatch. Consequently, the overall hatchability rate exceeded 80 %. The IoS groups displayed lower hatchability compared to the intact and IoM treated groups (*p* = 0.03). Analysis of the occurrence of suffocated or weak eggs showed a significant difference among the treatment groups (*p* = 0.003). The intact group showed a higher incidence of suffocation and weakness, while the lowest number of dead chicks was recorded in the IoM group. Fig. 1 presents the total hatchability, along with the numbers of weak, suffocated, and bloody or infertile eggs.

### Performance results

Table 5 summarizes the hatching weight results based on whether the eggs were treated *in ovo.*

**Table 5:** Hatching weights of birds assigned to experimental groups.

Treatment group	Intact	IoS	IoM
**Female**	46.3	46.8	47.4
**Male**	46.8	44.1	47.1
**SD**	0.9	1.2	1.4
**Treatment effect p-value**	0.22		
**Sex-effect p-value**	0.26		
**Interaction p-value**	0.28		

* Intact group includes:C1: positive control, ID: intact control group, Hyd: treatment group supplemented with Hydrogel®, HydM: treatment group supplemented with Hydrogel® and methionine.

*Hatching weights represent estimated marginal means of the groups.

The effect of the treatment (entire, *in ovo* saline or *in ovo* Met; *p* = 0.22), and the sex of the birds (*p* = 0.26) on hatching weight was insignificant. There was no interaction between the main effects (*p* = 0.28). The multiple factorial results on body weight of birds in different experimental group are presented in Table 6.

**Table 6:** Liveweight of the birds after feed deprivation, and on days 3, 10, 21, and 35.

**Day of measurement**	**LW3(g)**																		**LW10 (g)**												
**Treatment**	**C1**		**C2**			**ID**				**IoS**		**IoM**			**Hyd**			**HydM**	**C1**		**C2**	**ID**			**IoS**		**IoM**		**Hyd**		**HydM**
Female	57.1		56.4			41.8				41.7		43.4			42.8			42.4	229.4		220.5	193.8			190.6		194.0		183.9		187.2
Male	57.2		54.7			43.1				41.0		42.7			43.0			42.5	227.2		222.7	199.0			190.0		198.0		191.1		194.4
**SD**	2.2		2.1			0.6				0.5		0.7			0.7			1	4.5		7.9	5.7			6.9		6.3		9.7		10.3
**Effect of sex**	0.61																		0.07												
**Effect of treatment**	**<0.001**																		**<0.001**												
**Tukey test**	**c**		**b**			**a**				**a**		**ab**			**ab**			**a**	**b**		**b**	**a**			**a**		**a**		**a**		**a**
**Interaction**	0.14																		0.69												
**Day of measurement**	**LW21 (g)**																		**LW-35**												
**Treatment**	**C1**	**C2**		**ID**			**IoS**				**IoM**		**Hyd**			**HydM**			**C1**	**C2**			**ID**	**IoS**		**IoM**		**Hyd**		**HydM**	
Female	742.3	717.2		674.2			638.7				660		634.9			635.1			1550.75	1492.4			1411.85	1408.1		1422.1		1372.3		1380.6	
Male	758.1	723		665.2				611			680.1		643.2			674.7			1591.7	1546			1446.56	1385.7		1488		1422.7		1464.2	
**SD**	21.8	42.2			31.0			88.0			39.5		32.6			40.1			44.2	124.3			57.2	120.0		111.0		83.0		107.8	
**Effect of sex**	0.25																		**0.013**												
**Effect of treatment**	**<0.001**																		**<0.001**												
**Tukey test**	**c**	**b**			**ab**				**a**			**ab**		**a**			**ab**		**c**	**b**			**ab**	**a**		**ab**		**a**		**ab**	
**Interaction**	0.22																														

C1: positive control, C2: control group with in-ovo saline, ID: intact control group, IoS: in-ovo saline, IoM: in-ovo methionine, Hyd: treatment group supplemented with Hydrogel®, HydM: treatment group supplemented with Hydrogel® and methionine, LW21, LW35: live weight on days 21 and 35, respectively.

In groups C1 and C2, where the birds were fed right after hatching, the C1 group exhibited the highest weight on day 3, followed by the group C2 with a significant difference of 1.6 grams(*p* < 0.001). This trend was also observed in the delayed groups, as IoS had lower body weight than all the other groups. The in-ovo methionine treatment did not affect the body weight of the birds in the starter period compared to the delay-fed counterparts. There was also no significant difference between the IoM group, and the saline-or hydrogel-treated ones in the grower and finisher phase, it only differed from the control groups (*p* < 0.001). In addition, hydrogel supplements with or without methionine did not result in higher body weight than the other delayed groups. The treatment effect on body weight was significant throughout the study (*p* = 0.001) but the difference was confirmed between the chicken from entire, non-treated eggs and immediately fed control (C1) and the post-hatch starved groups. At the same time, the sex of the birds was counted as a substantial effect only at the end of the experiment (*p* = 0.013), where roosters were heavier with 50 grams.

Table 7 shows the ADG in the examined periods.

**Table 7:** Average daily gain of birds in the examined period.

Day of measurement	ADG 3-10														ADG 11-21												
**Treatment**	**C1**	**C2**		**ID**		**IoS**			**IoM**		**Hyd**	**HydM**			**C1**	**C2**		**ID**		**IoS**		**IoM**		**Hyd**		**HydM**	
Female	18.3	17.3		14.8		14.4			14.7		13.7	14.1			46.6	45.2		43.7		40.7		42.4		41.0		41.0	
Male	18.0	17.4		15.2		14.5			15.1		14.4	14.8			48.2	45.6		42.4		38.3		43.8		41.1		43.7	
SD	0.4	0.9		0.6		0.7			0.7		0.9	1			1.7	3.6		2.5		7.5		3.3		2.5		2.8	
**Effect of sex**	0.08														0.43												
**Effect of treatment**	**<0.001**														**<0.001**												
**Tukey test**	**d**	**c**		**b**		**d**			**b**		**a**	**ab**			**d**	**c**		**b**		**a**		**b**		**a**		**ab**	
**Interaction**	0.07														0.12												
**Day of measurement**	**ADG 22-35**														**ADG 1-35**												
**Treatment**	**C1**		**C2**		**ID**		**IoS**	**IoM**		**Hyd**			**HydM**		**C1**		**C2**		**ID**		**IoS**		**IoM**		**Hyd**		**HydM**
Female	57.6		55.5		52.7		55.6	55.1		52.8			53.3		42.9		41.3		39.0		38.9		39.3		37.9		38.1
Male	59.5		59.2		55.8		55.8	58.8		56.5			58.1		44.1		42.8		40		38.3		41.2		39.3		40.5
SD	3.8		6		2.7		5.3	4.4		7.2			5.7		1.3		3.6		1.6		3.4		3.2		2.4		3.1
**Effect of sex**	**<0.001**														**0.013**												
**Effect of treatment**	**0.013**														**<0.001**												
**Tukey test**	**c**		**b**		**a**		**b**	**b**		**a**				**ab**	**a**		**ab**		**c**		**c**		**bc**		**c**		**c**
**Interaction**	0.12														0.82												

C1: positive control, C2: control group with in-ovo saline, ID: intact control group, IoS: in-ovo saline, IoM: in-ovo methionine, Hyd: treatment group supplemented with Hydrogel®, HydM: treatment group supplemented with Hydrogel® and methionine, LW21, LW35: live weight on days 21 and 35.

There was significant treatment effect in growth rate of the birds (*p* < 0.001), the lowest ADG between days 3 and 10 was experienced in the Hyd group compared to the other experimental groups except HydM. At the same time, the sex of the birds had no effect in the starter phase (*p* = 0.08). This tendency was also true for the ADG between days 11 to 21. From day 22 to 35 only the ID and Hyd groups differed from the positive control (immediately fed) groups (*p* = 0.013), and roosters had higher ADG than pullets (*p* < 0.001). Among the delayed fed groups it can be stated that *in ovo* Methionine supplementation resulted the best growth rate in general. There was no difference in ADG between the immediately fed (C2) and the IoM group during the finisher phase, and the advantage of this treatment resulted statistically the same growth rate in the whole trial compared to the immediate fed *in ovo* saline treated counterparts.

Table 8 represents the feed intake (**FI)** and the feed conversion ratio (**FCR**) during the study.

**Table 8:** Effect of different early feeding methods on feed intake and feed conversion ratio.

**Treatment group**	**FI 1-3**	**FI 3-10**	**FI 11-21**	**FI 22-35**	**FI 1-35**	**FCR 3-10**	**FCR 11-21**	**FCR 22-35**	**FCR 1-35**
**C1**	7.09**a**	23.27**a**	70.65**a**	123.97	76.46**a**	1.47**a**	1.70	2.42	2.07
**C2**	4.58**a**	22.03**a**	68.83**ab**	120.2	74.14**ab**	1.47**a**	1.73	2.34	2.04
**ID**	-	18.14**b**	64.68a**b**	116.88	70.64**bc**	1.38**b**	1.72	2.38	2.04
**IoS**	-	17.71**b**	62.76**b**	115.25	70.47**bc**	1.37**b**	1.84	2.26	2.03
**IoM**	-	18.22**b**	63.77**ab**	118.75	70.28**bc**	1.39**ab**	1.69	2.44	2.03
**Hyd**	-	17.19**b**	62.18**b**	116.9	69.20**c**	1.41**ab**	1.73	2.44	2.01
**HydM**	-	18.02**b**	63.0**b**	118.9	69.03**c**	1.4**ab**	1.68	2.4	2.00
**P-value**	**<0.001**	**<0.001**	**0.002**	0.19	**<0.001**	**0.001**	0.52	0.65	0.83
**RMSE**	0.89	0.72	4.13	0.7	1.53	4.4	0.72	0.77	0.07

*Trt: Treatment groups, FI: feed intake, FCR: feed conversion ratio, C1: positive control, control group with in-ovo saline, ID: intact control group, IoS: in-ovo saline, IoM: in-ovo methionine, Hyd: treatment group supplemented with Hydrogel®, HydM: treatment group supplemented with Hydrogel® and methionine.

Feed intake differed among treatment groups from day 1 to 21. Between days 1 and 10, the FI of the C1, and C2 groups was higher (*p* < 0.001) than the birds with delayed feed, confirming that chickens could not compensate for the feed deprivation during the starter period. However, from day 11, the feed intake of the IoM group only differed from the intact control group (Ci vs IoM, *P* < 0.05). As expected, total FI (days 1 to 35) was the highest in the control groups, followed by the ID and the *in ovo* treated groups. Interestingly, birds with gel supplements (Hyd, HydM) had lower feed intake during the experiment, however, the difference was not statistically reliable compared to other delay-fed groups. Looking at the FCR results, it can be stated that it was the best for the ID and IoS groups in the starter period, and those significantly differed from both controls (*p* = 0.001). Later the feed efficiency was the same in all treatments (*P* > 0.10).

### Blood smear results

Results of the blood smear evaluation on day 21 is summarized in Table 9.

**Table 9:** Blood smear results on day 21.

Treatment groups										
	Absolute value (Douglas et al., 2010)	2C1	C2	ID	IoS	IoM	Hyd	HydM	P-value	RMSE
**Leukocytes**	**25.19**									
1**HE**	7.19±3.39	10.83**b**	9.19**ab**	8.16**a**	7.28**a**	9.97**b**	9.44**ab**	8.98**ab**	**0.001**	7.28
**LYM**	13.86±1.58	11.49**a**	13.40**ab**	14.04**b**	14.00**b**	11.92**a**	12.07**a**	12.27**a**	**0.02**	8.55
**MON**	1.94±1.01	2.43	2.24	2.29	2.94	2.50	2.60	2.91	0.48	3.81
**EOS**	0.64±0.92	0.39**a**	0.33**a**	0.66**ab**	0.94**b**	0.78**ab**	1.07**b**	1.03**b**	**0.01**	2.17
**THR**	-*	14.35**c**	13.00**bc**	9.94**ab**	7.86**a**	9.70**ab**	9.43**ab**	11.25**ac**	**0.003**	3.12
**Total WBC count**		25.14	25.16	25.15	25.16	25.16	25.18	25.18	0.25	2.34

1 HE: heterophil granulocytes, LYM: lymphocytes, MON: monocytes, EOS: Eosinophil granulocytes, BAS: basophil granulocytes, THR: thrombocytes, WBC: white blood cell.

2 C1: positive control, C2: control group with in-ovo saline, ID: intact control group, IoS: in-ovo saline, IoM: in-ovo methionine, Hyd: treatment group supplemented with Hydrogel®, HydM: treatment group supplemented with Hydrogel® and methionine, WBC: white blood cell ^3^a, b, and c letters represent significant differences.

⁎ -: No comparative data is available to define the number of thrombocytes.

The absolute number of white blood cell types was within the physiologically normal range (12 × 10^3^/μL to 30 × 10^3^/μL; Talebi et al., 2005). There was no difference between the treatment groups regarding the total white blood cell (**WBC)** count. No comparative data was available to determine the thrombocyte count, thus, the absolute thrombocyte count of treatment C1 was considered a kind of reference during the study. The number of platelets was the lowest in the IoS group and the highest in the C1, C2, and HydM groups. Still, none of the differences point out any inflammatory reactions or thrombocytosis. The morphology of basophils is very similar to monocytes, and their number is low in general, thus determining the physiologically normal, absolute cell number is hard. A reduced number of heterophils in treatments ID and IoS may show a general decline in immune protection. Birds with in-ovo methionine treatment or hydrogel supplementation (IoM, Hyd, HydM) had similar cell counts compared to the immediately-fed groups (C1, C2). In our experiment, methionine-supplemented treatment (IoM, HydM) positively affected the immune system, demonstrated by the evaluated number of lymphocytes, monocytes, and eosinophils compared to the C1 group.

On day 35, the leukogram (Table 10) resembled that of an adult chicken, except for the monocytes, as noted by Campbell (2012).

**Table 10:** Blood smear results on day 35.

				Treatment groups								
	Absolute value (Douglas et al., 2010)			C1	C2	ID	IoS	IoM	Hyd	HydM	P-value	RMSE
**Leukocytes**		**25.19**										
**HE**		7.19±3.39		9.02	9.51	8.65	9.72	9.26	7.33	9.13	0.07	7.94
**LYM**		13.86±1.5		12.57	12.72	12.20	12.18	12.70	13.60	12.57	0.63	8.73
**MON**		1.94±1.01		2.20**ab**	2.02**ab**	3.25**c**	1.74**a**	1.89**a**	2.77**b**	1.83**ab**	**<0.001**	3.26
**EOS**		0.64±0.92		0.94	1.09	1.55	1.34	1.34	1.66	1.76	0.25	3.53
**THR**		*-		8.84	9.33	7.96	8.67	9.13	9.48	9.33	0.71	11.18
**Total WBC count**				24.7	25.3	25.7	26.0	25.2	25.4	25.3	0.68	6.78

^1^HE: heterophil granulocytes, LYM: lymphocytes, MON: monocytes, EOS: Eosinophil granulocytes, BAS: basophil granulocytes, THR: thrombocytes.

^2^C1: positive control, C2: control group with in-ovo saline, ID: intact control group, IoS: in-ovo saline, IoM: in-ovo methionine, Hyd: treatment group supplemented with Hydrogel®, HydM: treatment group supplemented with Hydrogel® and methionine

^a,b,c^ letters indicate significant differences (*P* < 0.05).

⁎ -: No comparative data is available to define the number of thrombocytes.

The elevated number of this cell type in the ID group may refer to an activated immune response or distress (Nabity and Ramaiach, 2012). Our results are in line with Tombarkiewicz et al. (2020) reporting no negative effect of *in ovo* intervention, however, lack of positive effect of methionine either, since *in-ovo* administered folic acid nor methionine supplements affected the white blood cell counts on day 35 in their study.

### Lipid oxidation and antioxidative capacity

**Fig. 2:**
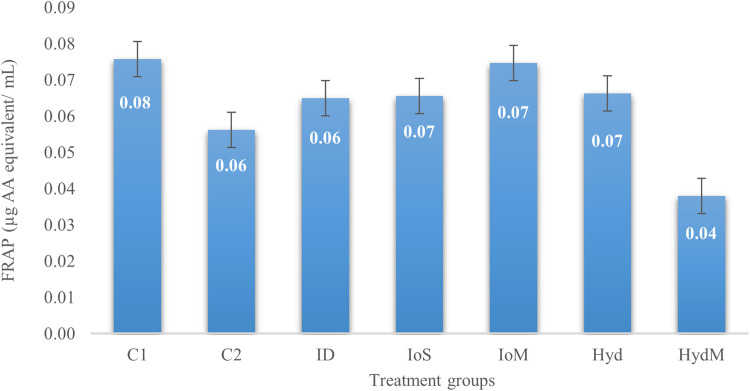
Total antioxidant capacity of blood plasma on day 21 (FRAP), *p* = 0.07.

**Fig. 3:**
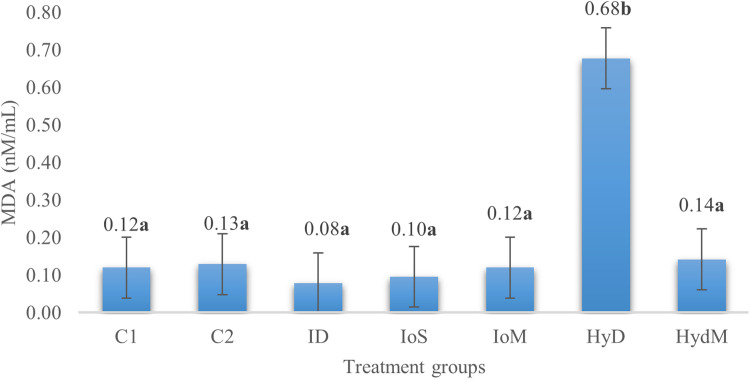
MDA levels of blood plasma on day 21, *p* = 0.07.

In our study (Figures 2 and 3), the total antioxidant capacity results showed no significant differences between the treatment groups (*p* = 0.07). On the other hand, a tendency shows that the immediately fed (C1, C2), or IoM groups had a slightly better antioxidant status than the HydM group. An interesting finding is, that FRAP values are higher in the ID group than in HydM. This can be the effect of chance, and seems difficult to explain. We found that injecting or feeding DL-Met does not reduce the antioxidant power compared to the control groups. Regarding the lipid oxidation, the significantly (2 times) higher value in Hyd group on day 21 should be an artifact, since it is unlikely that 3 weeks after consuming (for 2 days post-hatch) the Met enriched gel would have such a high impact, particularly because the housing and sample collecting conditions were the same in all groups.

Additionally, only 4 birds died during the experimental period in the entire study in different treatment groups, thus there was no physiological sign of extreme oxidative stress.

### Gene expression results of CYP450 2H1, IL-2 and IL-6

**Fig. 4:**
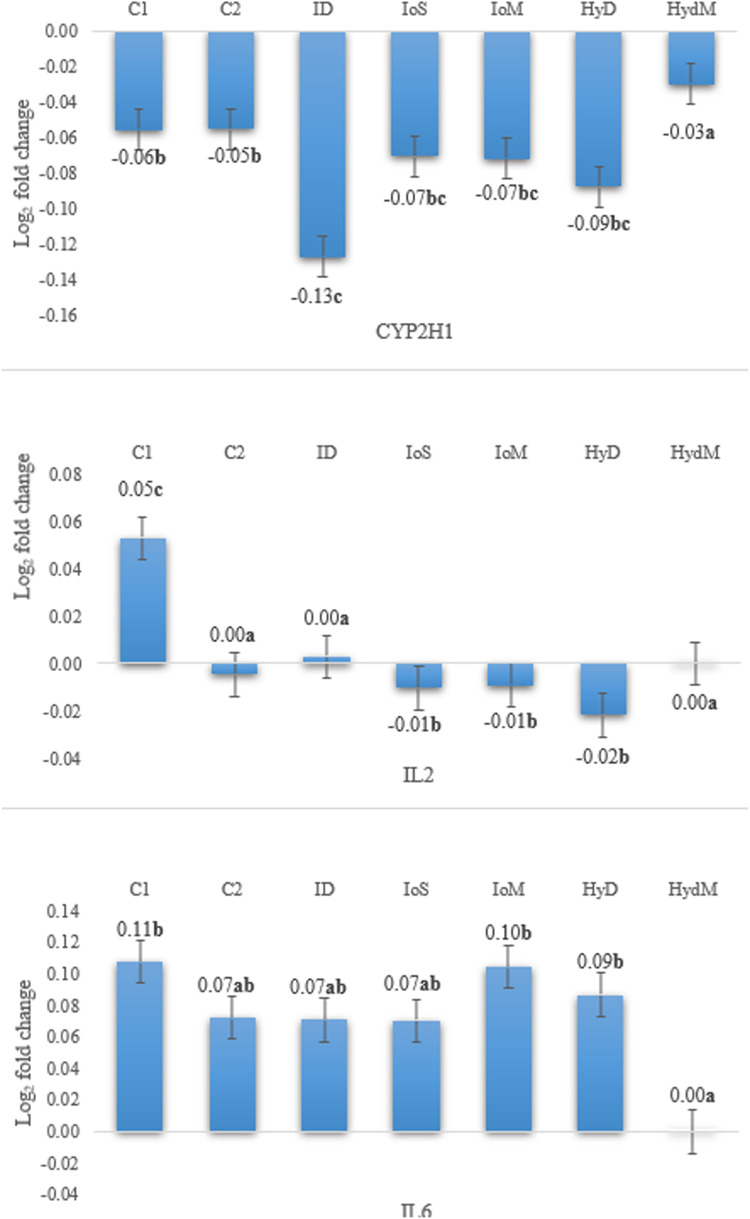
Relative expression CYP2H1, IL2, and IL6 genes in the liver on day 21 compared to the reference gene (Glycerynealdehyde-3-phosphate-dehydrogenase, GADPH), *p* < 0.001.

**Fig. 5:**
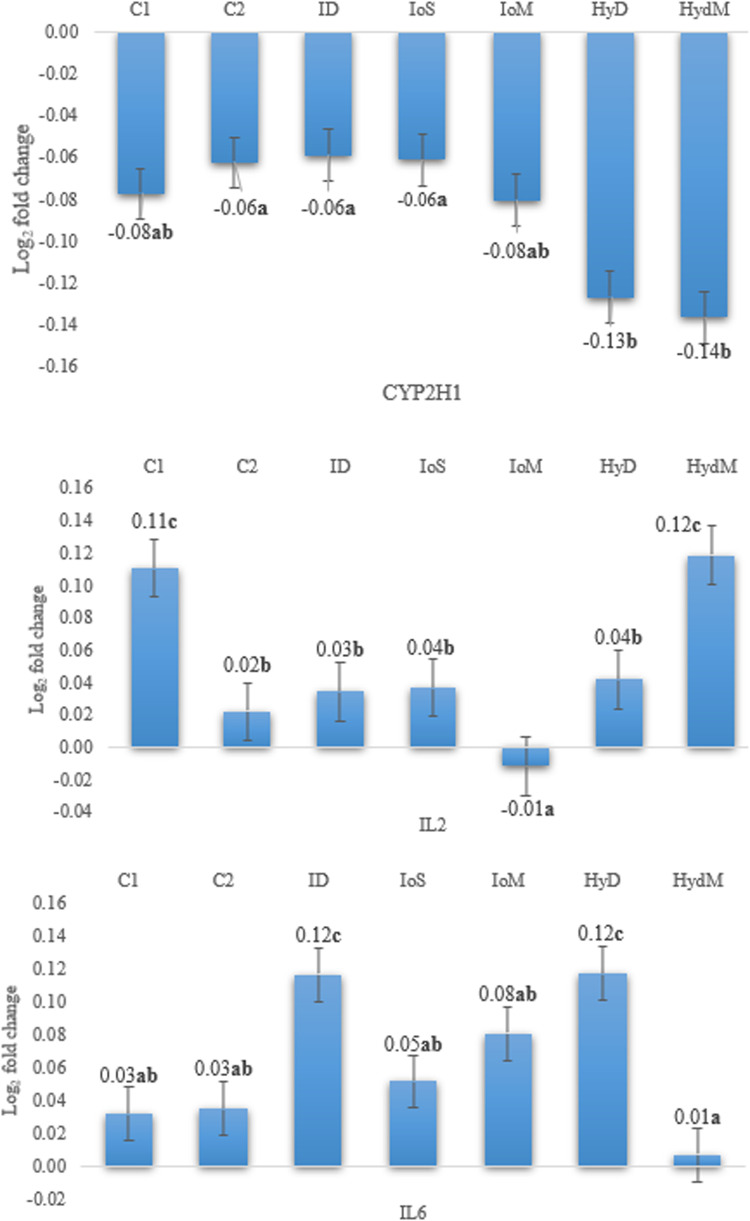
Relative expression CYP2H1, IL2, and IL6 genes in the liver on day 35 compared to the reference gene (Glycerynealdehyde-3-phosphate-dehydrogenase, GADPH), *p* < 0.001.

The gene expression results are presented in Figs. 4 and 5.

The relative expression of CYP2H1 compared to the housekeeping gene was negative in both time points, and the values were affected by the trial groups (*p* < 0.05). On day 21, the CYP2H1 gene expression was downregulated, the most in the ID group which differed from the C1, C2, and HydM counterparts (*p* < 0.001). The relative gene expression value obtained in C1 did not differ from any other treatment means, but there was a significant difference between C2, ID, IoS versus Hyd and HydM groups on day 35 (*p* < 0.001) and the HydM group showed the highest expression in the long run. The IL2 gene was upregulated in group C1 on day 21, but not in all other treatments (*p* < 0.001). On day 35, the IL2 gene was upregulated in all birds except for the IoM group (*p* < 0.001). In two groups, C1 (intact eggs and immediate feeding) and HydM (methionine-supplemented Hydrogel) IL2 had higher expression than in others (*p* < 0.05). All treatments in both time points upregulated the expression of IL6 except for HydM (HydM vs all others, *p* < 0.05), where the IL6 gene expression did not show any differences from the housekeeping gene.

## Discussion

Despite tremendous data, it is still unclear which specific conditions are responsible if *in ovo* intervention would influence the hatchability. There are many contradictive results on the effect of *in ovo* methionine injection on hatchability. According to Ohta et al. (1999) and Coskun et al. (2018), in-ovo DL-methionine decreased the hatchability in the early stages of incubation (days 0 to 7), on the contrary, McGruder et al. (2011) found that the effect of *in-ovo* methionine treatment on the number of dead or suffocated chicks is insignificant. These disagreements might result from the different injection techniques, the concentration of the solution, the date of injection, and the depth of the needle puncture. Kumagai et al. (2002) and Troen et al. (2003) reported that excessive methionine consumption can lead to developmental changes in the liver and the kidneys as the amount of a toxic metabolite (homocysteine) increases in the blood. Tombarkiewitz (2024) found increased homocysteine concentration by injecting 50 mg methionine/egg regardless of the injection day. Previous studies also reported that in-ovo manipulation -in general- decreased hatchability by 10 % to 35 % and rarely had positive or unbiased results as in some cases, it caused allergic reactions (Kocamis et al., 1998, Kocamis et al., 1999; Lamosová et al., 2003; Heiblum et al., 2001). Embryonic mortality can also be a result of an immature immune system. The individual variance in programming and development of the defense mechanisms may explain if *in ovo* intervention could be a perturbation in some cases while a support in others. Hincke et al. (2019) reported that the 18th day of incubation is critical because the embryos can activate the immune response against pathogens from this date. Thus, in our case, adding *in-ovo* nutrition on day 17 may cause a delay or modification in this process.

Increasing hatching weight is important from a practical point of view since it is an important proxy of market weight. According to Wilson (1991), a 1 g increase in weight at hatch can result in an 8-13 g increase in body weight at market age. In our study, the 1.6 g increase in hatching weight for group C1 led to a 52 g advantage at market age compared to group C2. Hatching weight was not affected by the *in ovo* manipulation in this study. Regarding perinatal Met supplementation, few studies report its benefits on hatching weight In contrast, there are more positive findings associated with in ovo carbohydrate feeding, as reviewed by Retes et al. (2018). Coskun et al. (2018) noted that in-ovo injections of methionine and lysine did not impact the relative weight of chicks. Similarly, Lugata et al. (2024) found that in-ovo application of DL-methionine did not affect chick weight compared to the intact control group, regardless of the genotype (intensive or slow-growing strains) of the birds. This finding contrasts with the results from Farias et al. (2023), which reported that in-ovo administration of DL-methionine led to higher hatching weights.

Research consistently shows that the growth performance of broilers during their first week is a strong indicator of their eventual slaughter weight, as well as the overall mortality and morbidity rates within the flock. As a result, farms implement early feeding methods to support the birds after hatch. One of the simplest methods is to provide a hydrogel upon their arrival at the barn or even earlier, while still in the transport box. Studies by Buenaventura (2022) and Özlü et al. (2022) have demonstrated that gel supplements with varying compositions and consistencies can have beneficial effects after hatching. These gel supplements not only offer additional hydration but also include probiotic supplementation during the holding period, potentially reducing cumulative mortality. Furthermore, research indicates that in ovo feeding provides early support to the birds with essential nutrients, delivering the carbohydrates they lack at hatching and supporting immune functions through amino acids. This method may also have epigenetic effects that influence the birds' overall lifespan. Coskun et al. (2014) reported that in-ovo methionine enhances average daily gain (ADG) after incubation. The embryo uses this in-ovo methionine for protein synthesis while preventing protein degradation, which contributes to better body weight development in the birds (Ohta and Kidd, 2001). Different studies have yielded varying results regarding growth performance in *in ovo* research. Bhanja et al. (2004), Al Darji et al. (2012), and Coskun et al. (2018) found that in-ovo injections of lysine and methionine did not significantly affect the birds' body weight (BW), feed intake (FI), feed conversion ratio (FCR), or average daily gain (ADG). In contrast, earlier studies indicated that administering amino acids in-ovo can improve these growth parameters. Furthermore, these studies suggested that factors such as the timing, injection depth, and concentration of the solution may have a greater influence on health and performance than the type of feed supplement used (Foye et al., 2006; Kadam et al., 2008; Shafey et al., 2014).

Upon reviewing the blood smear results, we found that, consistent with the findings of Latimer and Bienzle (2010), the increase in white blood cells (WBC) due to post-hatch stress decreases with age. Tombarkiewicz et al. (2020) reported that the in-ovo methionine-treated group exhibited higher WBC counts on day one, but this difference diminished over time. The slight variations in the counts of different cell types, compared to the reference range provided by Douglas et al. (2010), may be attributed to the different ages at which blood samples were collected. Our findings also highlight the long-term effects of post-hatch stress caused by starvation, particularly its negative impact on immune parameters. Notably, our data suggest that in-ovo methionine supplementation may help mitigate the distress associated with feed deprivation on the first day, as the counts of immune cells (heterophils, lymphocytes, and eosinophils) were statistically similar to those in the immediately fed control group. Additionally, according to Tombarkiewicz et al. (2020), platelet counts on day seven varied from 6.67 ± 2.5 in the in-ovo saline group to 13.75 ± 13.83 in the in-ovo folic acid group. In birds, heterophil granulocytes play a crucial role in phagocytosis (Cook, 2015), and their levels were consistently lower in delayed-fed birds unless methionine supplementation was provided in hydrogel form.

As enzymatic reactions are linked to defense mechanisms against oxidative stress, it is crucial to define the total antioxidant content. These compounds work together to neutralize reactive oxygen species. Factors such as temperature, humidity, diet, vaccination protocols, disease challenges, and the duration birds spend in their pens can all impact their antioxidant status (Surai, 2007). According to Khajali et al. (2010), higher FRAP (Ferric Reducing Antioxidant Power) values indicate a stronger antioxidant capability. Research suggests that the total antioxidant capacity can be enhanced by dietary supplementation with methionine (Liu et al., 2019), although it remains uncertain whether a single dose of methionine significantly influences this capacity. In our study, the results for total antioxidant capacity indicated no significant differences between the treatment groups (*p* = 0.07). However, there was a tendency for the groups receiving immediate feeding (C1, C2) or in ovo methionine (IoM) to show slightly better antioxidant status compared to the Hydrogel® group. This phenomenon might be attributed to the fact that in ovo administration occurs earlier than Hydrogel® feeding, potentially resulting in lower supplementation intake during transport. Other findings suggest that the antioxidant capacity of plasma is highly dependent on the type of methionine used (L-methionine vs. DL-methionine), the dosage, the age of the animals following in ovo or early nutrition treatment, and their interactions (Jankowski et al., 2018). Kim and Bayley (1983) and Lugata et al. (2024) demonstrated that L-methionine may be more effective than DL-methionine due to its better utilization, indicating it could be more efficiently used in future studies.According to Nielsen et al. (1997), the MDA levels are related to the degree of cell damage during lipid oxidation. Although, Met is indispensible for gluthation peroxidase enzyme production, high methionine diet could induce oxidative stress and increase serum MDA (Yalçinkaya-Demirsöz et al., 2009). However, Lugata et al. (2024) found that different genotypes may respond differently to in-ovo methionine supplemtation as regards their antioxidant status. In our study MDA was not affected by early provision of Met, witch do not conflict with the findings that in-ovo methionine supplements may result in lower plasma MDA under heat-stress conditions in the finisher phase (Yeiha et al., 2024).

In the first week of life, leukocyte populations expand to populate the lymphoid organs, which are crucial for immune response later in life (Fellah et al., 2014). During this time, certain regulatory peptides function as extracellular signals, aiding in immune responses (Cohen et al., 1974). Key families of these cytokines include interleukins (IL), interferons (**IFN**), tumor necrosis factor (**TNF**), transforming growth factor (**TGF-β**), colony-stimulating factors (**CSF**), and chemokines. T-cells produce IL-2, which is significant for avian-mammal similarities, including natural killer (**NK**) cell activation, lymphocyte proliferation, and the clearance of pathogens from the body (Stepaniak et al., 1999; Staeheli et al., 2001). Interleukin-6 (IL-6) is produced by various cells, including hepatocytes, hematopoietic progenitor cells, T-cells, B-cells, and cells of the central nervous system. It plays a vital role in hematopoiesis and the regulation of immune responses (Hirano et al., 1986; Ikebuchi et al., 1987; Houssi Kishimoto et al., 1995). The current gene expression profiling was used to determine the immunomodulation process in the liver on the long run, as it has the strongest expression of CYP1-3 superfamily enzymes based on previous studies of tissue-specific distribution patterns (Jönsson et al., 2011). The liver was also reported as the most sensitive organ to methionine deficiency (Klünemann, 2024) and is known as a detoxifying organ by eliminating toxins, xenobiotics, and oxidative stress (Sharma and Dubey, 2007).

CYP2H1 is a member of the CYP 1-3 enzyme superfamily, which plays a role in helping cells eliminate stress reactions (Yehia et al., 2002). It is also correlated with the protein levels of mRNA expression (Ohtsuki et al., 2011; Temesvári et al., 2012). All groups displayed moderate expression of the examined genes, which aligns with the results from the FRAP analysis. This indicates that the total antioxidant capacity values were adequately elevated and remained stable, showing no significant differences. The expression levels of CYP2H1 in the ID group differed from those in the positive control (C1) and the HydM group, suggesting slower protein utilization relative to the average daily gain (ADG) results. Klünemann et al. (2024) found that methionine plays a crucial role in liver function, as a deficiency in this amino acid leads to reduced expression of genes involved in nutrient metabolism within the liver. The negative fold change values of CYP2H1 indicate a decline in gene expression during the examined period. This finding is consistent with Waterland (2006), who noted that methionine supplementation could increase DNA methylation of genes, leading to downregulation of mRNA expression. However, such effects are not always predictable, as methylation occurs only at specific loci (Amaral et al., 2011).

Avian IL-2 is part of the Th2 interleukin superfamily and plays a crucial role in inducing effector cytokines, as well as targeting effector cells such as B cells, eosinophils, and basophils during humoral immune responses (Henderson et al., 1999). The observed downregulation of IL-2 in the IoS, IoM, and HyD groups compared to the C1 group suggests that the liver becomes more tolerant to antigens entering through the hepatic portal blood from the gut (Wishna-Kadawarage et al., 2025). Introducing symbiotics into broiler diets during the embryonic stage may produce effects similar to those seen in immune-related genes. Dunisławska et al. (2023) reported significant downregulation of spleen-associated tyrosine kinase (SYK) and kelch-like family member 6 (KLHL6) in the liver.

The upregulation of IL-6 observed during the trial—particularly with significantly higher log2 fold change values in the C1 and IoM groups on day 21—may indicate beneficial immunomodulatory effects, especially in early life. Previous studies by Kogut et al. (2003) and Philbin et al. (2005) reported increased expression of pro-inflammatory cytokines, leading to an active oxidative burst. Additionally, Shini et al. (2010) found that mRNA upregulation of IL-6 is strongly correlated with heterophil counts in chickens, which is consistent with our results on day 21. In a different study, Yarru et al. (2009) demonstrated that adding the immunosuppressive aflatoxin B1 to the diet at a dose of 1 mg/kg resulted in interleukin downregulation, which made the birds more susceptible to secondary bacterial and viral infections. Additionally, IL-6 has multiple functions, including promoting the differentiation of B cells into antibody-producing plasma cells, facilitating the growth and differentiation of T cells as well as leukemic cell lines into macrophages, aiding in the maturation of megakaryocytes, and stimulating acute phase protein synthesis in hepatocytes (Kaspers et al., 2021).

Despite these findings, the molecular mechanisms behind these changes are poorly understood. Therefore, we can only speculate that early administration of DL-methionine may have beneficial immunomodulatory effects such as immediate feeding.

## Conclusions

Ideal conditions for meat-type chickens are rarely achieved in practice, which can negatively impact their growth and immune competence. Based on the results of this experiment, the early administration of dietary methionine positively affects performance; however, none of the treatment groups could compete with immediate feed access. The gene expression results support the idea that *in ovo* supplementation of methionine has a long-term epigenetic effect, particularly concerning anti-inflammatory regulation. Additionally, the time lag between hatching and the first feeding increases the number of heterophils and monocytes, which may be a stress response that affects overall lifespan. Nevertheless, early application of dietary methionine-especially *in ovo*-may enhance protein metabolism and immune protection. Further studies are needed to investigate the potential benefits of early nutritional treatments and the dynamic relationship between nutrition and gene expression throughout the avian lifetime.

## Declaration of competing interest

The authors declare the following financial interests/personal relationships which may be considered as potential competing interests:

Veronika Halas reports financial support was provided by Government of Hungary, under the Economic Development and Innovation Operational Programme (GINOP). Veronika Halas reports article publishing charges was provided by Hungarian University of Agriculture and Life Sciences, Flagship Research Programme. If there are other authors, they declare that they have no known competing financial interests or personal relationships that could have appeared to influence the work reported in this paper.
